# Behavior of Active Polymer Knots

**DOI:** 10.1021/acs.macromol.5c01381

**Published:** 2025-10-07

**Authors:** Zhiyu Zhang, Longfei Li, Yongjian Zhu, Rui Zhang, Mingcheng Yang, Liang Dai

**Affiliations:** † Department of Physics, 428688 City University of Hong Kong, Hong Kong 999077, China; ‡ Beijing National Laboratory for Condensed Matter Physics and Laboratory of Soft Matter Physics, Institute of Physics, 47849 Chinese Academy of Sciences, Beijing 100190, China; § Department of Physics, 162635 Hong Kong University of Science and Technology, Hong Kong 999077, China

## Abstract

We investigate active polymer knots using Brownian dynamics simulations. We find the interplay of active force, chain connectivity, and knotting leads to several unexpected phenomena. First, active force significantly tightens knots through activity-induced stretching effect. The magnitude of the stretching effect differs greatly in and out of the knot core, probably because knotting modifies the arrangement of monomers and thus affects the stretching effect. We develop an approximate theory to quantify the dependence of the knot size on Péclet number *Pe*, which describes the activity strength. Second, active polymer knots significantly differ dynamically from nonactive polymer knots under tension. For example, active polymers exhibit knot breathing, i.e., switching between a very loose knot and a very tight knot, which is absent in nonactive knot under tension. Third, activity can shrink the conformations of very short chains, and knotting appears to enhance this activity-induced shrinkage. Fourth, in long knotted chains, activity-induced shrinkage vanishes because activity can reallocate segments from the knotted to the unknotted portion. This reallocation enlarges the overall conformation, counteracting the shrinkage effect. These results may have biological implications, considering that active force, chain connectivity, and knotting exist in biopolymers, such as DNA.

## Graphical abstract

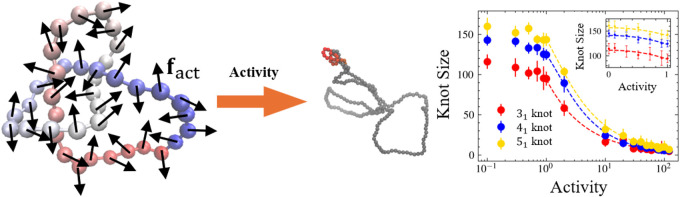

## Introduction

Active matter has gained extensive research interests in the past years because of two motivations. First, adding active force into Brownian particles can lead to many new interesting physical phenomena, such as motility-induced phase separation.^1,2^ Second, active force widely exists in biological systems, and the investigation of active matter can help understand the collective behavior in biological systems, such as swarming.^3,4^

Adding chain connectivity into active Brownian particles results in active Brownian polymers, which exhibit new phenomena due to the interplay of chain connective and active force.^5–19^ Active polymers have been experimentally realized using synthetic chains of active self-propelled Janus particles or oil droplets.^20,21^ Simulation studies found active polymer may swell or collapse depending on the strength of the active force.^8,10,11,14,15,22–27^ Theoretical works focus on analyzing solutions to the equation of motions of active polymer.^28^ Active polymers are also biologically relevant. In recent studies, activity has been linked to the compartmentalization of chromosomal DNA.^29,30^ Experiments of active polymer-like worms revealed that activity can strongly affect the tangling and untangling of worms.^31,32^

Recent attention has turned toward the topological aspects of active matter systems. In active fluids, topological defects have been observed to spontaneously move or follow predetermined paths.^33,34^ Remarkably, active topological waves, characterized by band structures, display resilience against obstacles and boundary effects.^35^ Inspired by the intriguing phenomena induced by chain connectivity and active force, we are curious about how knotting, as a topological constraint, is affected by chain connectivity and activity in active knotted ring polymers. Knotting is common for chain-like objects, including macroscopic ropes and polymers.^36,37^ Knotting may affect the ring configuration through the pathway that knotting modifies the spatial arrangements of monomers (Brownian particles), which affects the collective behavior of active Brownian particles. Some recent studies also find unusual self-knotting behaviors in active open chain polymers.^38,39^ Active knotted polymers are also biologically relevant, considering that DNA and proteins also experience active force and knotting during replication and transcription.^29,30,40,41^

Driven by the motivation to understand the interplay between activity and topology in polymers, we investigate active polymer knots using Brownian dynamics simulations. Our study reveals a strong knot localization mechanism in active ring polymer systems, which we explain by developing a theoretical framework that maps this behavior to an effective stretching response induced by activity. This analysis highlights how activity influences topological constraints in such systems. Interestingly, we also find that while activity promotes knot localization, the presence of knots in turn suppresses the activity-induced polymer shrinkage observed at low activity levels. The Methods Section details our active Brownian polymer model, including simulation and analytical techniques, while Knot Analysis Section outlines our knot analysis approach. The Results and Discussion Section presents findings on activity-induced knot localization (Strong Knot Localization section), theoretical analysis (Approximate Theory for Activity-induced Knot Tightening section), and the suppression of shrinkage by knotting (Dynamics of Knot Tightening by Activity section). We conclude with a discussion of our results in Conclusion section.

## Model and Methods

### Model and Molecular Dynamics Simulation

We consider a flexible and knotted polymer ring comprising monomer beads of diameter σ. With the simplification of neglecting hydrodynamic effects, our choice of dynamics is akin to an active version of the Rouse Model, where all Brownian beads are connected via springs^42^ but propelled by uncorrelated active forces **f**_act_ (Figure 1a). The magnitude of the active force acting on each bead remains constant, with its direction subject solely to rotational diffusion. The resulting equation of motion for bead i in three dimensions reads as

1 $$\gamma \frac{dr_{i}\left(t\right)}{dt}=-\sum_{j}\nabla V_{\mathrm{tot}}\left(r_{i}-r_{j}\right)+f_{i}^{r}\left(t\right)+f_{\mathrm{act},i}$$

where γ is the viscous drag coefficient, **f** *_i_^r^* is the Gaussian random force that averages zero and follows ⟨*f*_α,*i*_ *^r^* (*t*), *f*_β,*j*_ *^r^* (*t*′)⟩ = 2*k*_B_ *Tγδ*_*ij*_δ_αβ_δ­(*t* – *t*′). The last term in the equation of motion is the active force **f**_act,*i*_ = f_a_ **û**_i_, where **û**_i_ is the orientation of the particle which undergoes rotational diffusion

2 $$\gamma_{r}\frac{d{û}_{i}\left(t\right)}{dt}={û}_{i}\times \Lambda_{i}^{r}$$

with ⟨Λ_α,*i*_^r^(*t*), Λ_β,*j*_ *^r^* (*t*′)⟩ = 2*k*_B_ *Tγδ*_*ij*_δ_αβ_δ­(*t* – *t*′). We define the rotational diffusion constant as *D*_*r*_ = 3*D*_*t*_, where *D*_*t*_ is the translational diffusion and is given by *D*_*t*_ = *k*_*B*_T/γ.

**Figure 1:**
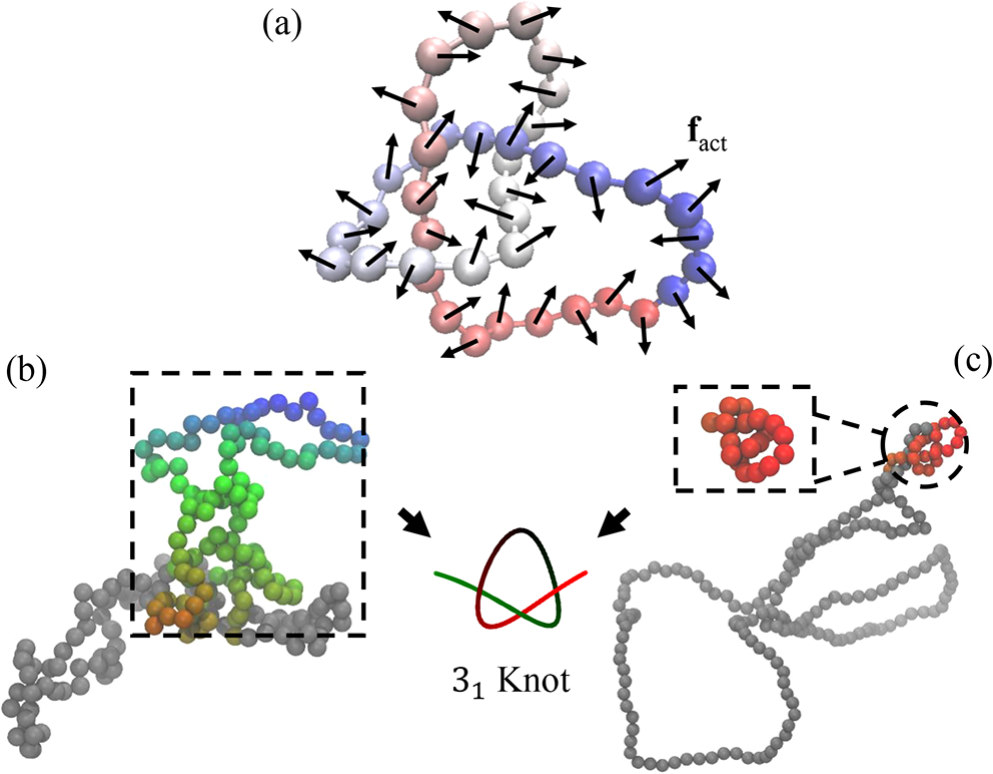
Simulation model. (a) Illustration of a knotted polymer ring. The arrows represent the active force **f**_act_ on beads. (b) A simulation snapshot of our simulation with the chain length of *L* = 200 and *Pe* = 0, i.e., activity turned off. The colored beads indicate the region of the knot core. (c) A simulation snapshot of our simulation with the same chain length but at *Pe* = 120. The knot core is shrunk into a small region indicated by red beads. The knot type here is 3_1_ using the Alexander*-*Briggs notation, where 3 is the minimum crossing number and 1 is an index to distinguish the knot types with the same minimum crossing number.

In our simulations, the Weeks–Chandler–Anderson potential describes the bead–bead interaction, i.e., the purely repulsive Lennard-Jones potential. We apply the finite-extensible nonlinear (FENE) spring for the bond interaction between adjacent beads. The net potential *V*_tot_ = *V*_FENE_ + *V*_excl_ has two parts, where the first part is from FENE potential *V*_FENE_

$$V_{FENE}=-15\epsilon \frac{r_{0}^{2}}{\sigma^{2}}\;\ln\left(1-{\left(\frac{r}{r_{0}}\right)}^{2}\right)$$

with *r*_0_ = 1. 5σ and ϵ = 1. *V*_excl_ is the WCA exclusion potential among non-neighboring beads

3 $$V_{\mathrm{excl}}=\{\begin{array}{ll} 4\epsilon \left[{\left(\frac{\sigma }{r}\right)}^{12}-{\left(\frac{\sigma }{r}\right)}^{6}\right]+\epsilon & r<2^{1/6}\sigma \\ 0 & \mathrm{otherwise} \end{array}$$

The FENE and WCA interactions can prevent the interpenetration of segments and ensure that the knot type of the polymer ring is preserved during simulation^43^ (see Supporting Information). We adopt the Péclet number to quantify activity, denoted as *Pe* ≡ *f*_act_σ/*k_B_T*, where σ is the bead diameter, *k*_*B*_ is the Boltzmann constant, and *T* is the temperature. We have adjusted *Pe* by two approaches: (i) varying *f*_act_ while fixing *T*; (ii) varying *T* while fixing *f*_act_. Both approaches give similar results (see Supporting Information). One limitation of the first approach is that a large *f*_act_ can cause simulation instability, as bond overstretches due to large moving distances of beads in a single step, resulting in topology being broken. This problem might be alleviated by reducing the time step, but a small time step reduces the simulation efficiency. Eventually, we select the second approach to explore a wide range of *Pe* ∈ [0, 120]. Note that our model is different from the one in the simulations of active polymer melts by Chubak et al.^44^ In the study by Chubak et al., activity arises from heterogeneous temperature along the polymer, whereas in our work, activity is driven by a randomly rotating active force.

The simulation results presented in the main text are for the chain length of 200 beads. We run simulations for 10^9^ steps with step size Δ*t* = 10^–4^τ_*r*_ to 10^–6^τ_*r*_. We provide several movies to better see the active rings in real-time at different activity levels (see Supplementary Movies). The simulation results for polymer rings with different lengths exhibit similar behavior (see Supplementary).

### Analysis of the Equation of Motion (EOM)

To analyze the conformational properties of the active Brownian rings and map the activity to an effective stretch, we can rewrite eq 1 as the following continuum dynamical equation of motion by ignoring nonlinear steric effects among beads

$$\gamma \frac{dr_{i}\left(t\right)}{dt}=k\frac{\partial^{2}r_{i}}{\partial i^{2}}+f_{i}^{r}\left(t\right)+f_{\mathrm{act},i}$$ 4

with the ring closure condition **r**_0_ = **r**_*N*_ and **r**_*N*+1_ = **r**_1_. The term $k\frac{\partial^{2}r_{i}}{\partial i^{2}}$ corresponds to the harmonic bond, which is different from the FENE bond in our simulations. The reasons we use this term are that first-order Taylor expansion of the FENE is same as the harmonic bond; the harmonic bond allows the analytical calculation. Following previous studies by others,^10,42^ we perform the following coordinate transformation

5 $$X_{p}\left(t\right)=\int_{0}^{N}\phi_{\mathrm{pi}}r_{i}\left(t\right)di$$

6 $$\phi_{\mathrm{pi}}=\frac{1}{N}\;\cos\left(\frac{p\pi i}{N}\right)$$

where *p* is an even number. Then, eq 4 can be transformed from the second-order stochastic differential equation (SDE) into an Orstein-Ulenbeck-type SDE

7 $$\begin{array}{l} \gamma_{p}\frac{\partial X_{p}\left(t\right)}{\partial t}=-k_{p}X_{p}\left(t\right)+{\mathrm{f̃}}_{p}^{r}\left(t\right)+{\mathrm{f̃}}_{\mathrm{act},p},\\ k_{p}=\frac{k\gamma_{p}}{\gamma }{\left(\frac{p\pi }{N}\right)}^{2} \end{array}$$

and **f̃**_p_^r^(t) and **f̃**_act,*p*_ are

8 $$\begin{array}{l} {\mathrm{f̃}}_{p}^{r}\left(t\right)=\frac{\gamma_{p}}{\gamma }\int_{0}^{N}\phi_{\mathrm{pi}}f_{i}di,\\ {\mathrm{f̃}}_{\mathrm{act},p}=\frac{\gamma_{p}}{\gamma }\int_{0}^{N}\phi_{\mathrm{pi}}{\mathrm{f̃}}_{\mathrm{act},i}di \end{array}$$

We choose γ_*p*_ = 2*Nγ* following previous literature.^42^ The backward coordinate transformation gives

9 $$r_{i}\left(t\right)=2\sum_{p=1}^{\infty }\phi_{\mathrm{pi}}X_{p}\left(t\right)\cdot \left(p\in \mathrm{even}\right)$$

These equations can be used to obtain the average bond stretch *L*_*b*_ for the active ring polymers. Since *L*_*b*_ is the average length between neighboring beads, we have by using eq 9

$$\left\langle L_{b}^{2}\right\rangle =\left\langle \frac{1}{N}\int_{0}^{N}{\left|r_{n+1}\left(t\right)-r_{n}\left(t\right)\right|}^{2}dn\right\rangle =2\sqrt{2}\sqrt{\overset{\infty }{\underset{p=even}{\sum }}\langle X_{p}X_{p}\rangle \sin^{2}\frac{p\pi }{2N}}$$ 10

A lengthy derivation gives the expression for ⟨**X**_*p*_ **X**_*p*_⟩. Combining above equations, we can solve for the relation between average stretch and *Pe* as

11 $$L_{b}\equiv \sqrt{\left\langle L_{b}^{2}\right\rangle }∼\sqrt{1+C{\mathrm{Pe}}^{2}}$$

where *C* is some constant independent of activity. The detailed derivation that leads to eq 11 can be found in the Supporting Information.

### Knot Analysis

We identify the knots and calculate knot sizes on ring polymers using the same procedure as our previous studies.^45,46^ We first select evenly spaced sites along the chain to cut. Each cut site can convert a ring to an open chain. For the open chain, we calculate the knot core by removing beads one by one from both chain ends until the knot type changes. During the calculation, we use the Alexander polynomial and the minimally interfering closure scheme.^47^ The number of beads in the knot core is defined as the knot size, *L*_*k*_. Four cut sites yield four knot sizes, and we record the smallest one among these four values as the knot size for the ring.

## Results and Discussion

### Strong Knot Localization

Our simulations show that as activity increases, the knot core shrinks (Figures 1b,c and 2a). The colored beads in Figure 1b,c indicate the knot core. At *Pe* = 0, the knot core spreads over about 100 beads, i.e., about half of the polymer ring (Figure 1b). At *Pe* = 120, the knot core shrinks to 14 beads (Figure 1c). Note that *L*_*k*_ = 14 corresponds to a very tight knot considering the tightest trefoil knot core contains 11 beads.^48^ Figure 2a shows average knot-core size *L*_*k*_, i.e., the average number of beads in the knot core, as a function of *Pe* for three knot types. We offset *L*_*k*_ by the tightest knot size: *L*_*k*_^min^ = 11 for 3_1_ knot, *L*_*k*_^min^ = 14 for 4_1_ knot, *L*_*k*_^min^ = 17 for 5_1_ knot. With the increase of *Pe*, *L*_*k*_ – *L*_*k*_^min^ approaches zero, indicating the knots approach the tightest conformations.

**Figure 2:**
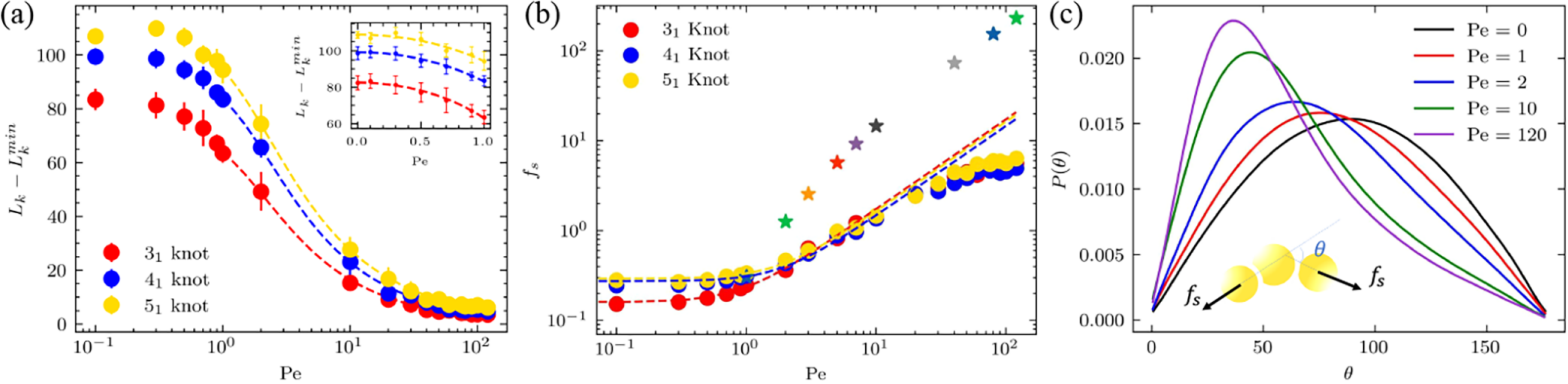
Knot tightening induced by active force. (a) The average number of beads in the knot core, *L*_*k*_, as a function of *Pe*. We offset *L*_*k*_ by the tightest knot size: *L*_*k*_^min^ = 11 for 3_1_ knot, *L*_*k*_^min^ = 14 for 4_1_ knot, *L*_*k*_^min^ = 17 for 5_1_ knot. Dash lines are from eq 13. The inset represents scaling in the low activity regime and the dash lines are from eq 14. (b) The effective stretching force on the knot estimated from the knot size-force relationship in nonactive-force case. Dash lines represent theoretical predictions based on eqs 13 and 14. The star symbols are the effective stretching forces estimated from the bending-angle distribution of ideal active polymers containing three beads. (c) Bending-angle distribution of ideal active polymers containing three beads.

Activity-induced stretching also manifests in terms of bending angle distribution. Figure 2c shows the distribution for an ideal three-bead active polymer. It is worth noting that for an ideal flexible chain without active force, the bending angle is not uniformly distributed between 0 and 180°. Instead, the probability of bending angle, θ, is proportional to sin­(θ), because the conformational space is proportional to sin­(θ) (see Supplementary). With an increase in *Pe*, the stretching effect becomes stronger, and the distribution shifts toward smaller bending angles. Using the relationship between stretching force and bending-angle distribution in nonactive polymers (see Supplementary), we can estimate the effective stretching force induced by activity from the bending-angle distributions in active polymers. The star symbols in Figure 2b shows the estimated effective stretching force induced by activity. Here, we use the active polymers as ideal active polymers containing three beads for the purpose of eliminating complex interplay of knotting and activity in active polymers and focusing on analyzing activity-induced stretching effect. Overall, the change in bending-angle distribution can serve as a “sensor” for the effective stretching force induced by activity.

Besides the bending-angle distribution, the change in knot size can also serve as a “sensor” for the effective stretching force induced by activity. Accordingly, we run equilibrium simulations without active forces to obtain the quantitative relationship between the stretching force and knot size.^49^ Using this relationship, we convert the knot size into an effective stretching force, *f*_*s*_, as shown by colored circles in Figure 2b. We note that although previous work has shown that dipolar extensile (DPE) and dipolar contractile (DPC) active polymers cannot be mapped onto effective equilibrium models with renormalized spring constants,^17^ our results demonstrate that our system exhibits an effective correspondence to stretched polymer knots.

Furthermore, the change in bond length can also serve as a “sensor” for the effective stretching force induced by activity. Intriguingly, we find that magnitudes of activity-induced bond stretching slightly differ inside and outside the knot core (Figure 3). Specifically, we compare three regions: the entire ring, the knot core, and the five beads immediately adjacent to the knot head (labeled as “Left” in Figure 3 and illustrated by the diagram in the middle). To isolate the impact of knotting on the stretching effect, we normalize the average bond length of the ring polymer by the value for an unknotted at the corresponding *Pe*. The stretching effect is stronger in the knot core than in other regions of the polymer. For the bonds in the vicinity of the knot core, the stretching effect is also stronger than in the areas far away from the knot core. It is possible that knotting modifies the spatial arrangement of beads in and around the knot core, producing entanglements or crossings which affects the collective behavior of the active forces on beads. Figure 3 shows the results for three different knot types, which indicates weak dependence of the stretching effect on the knot type.

**Figure 3:**
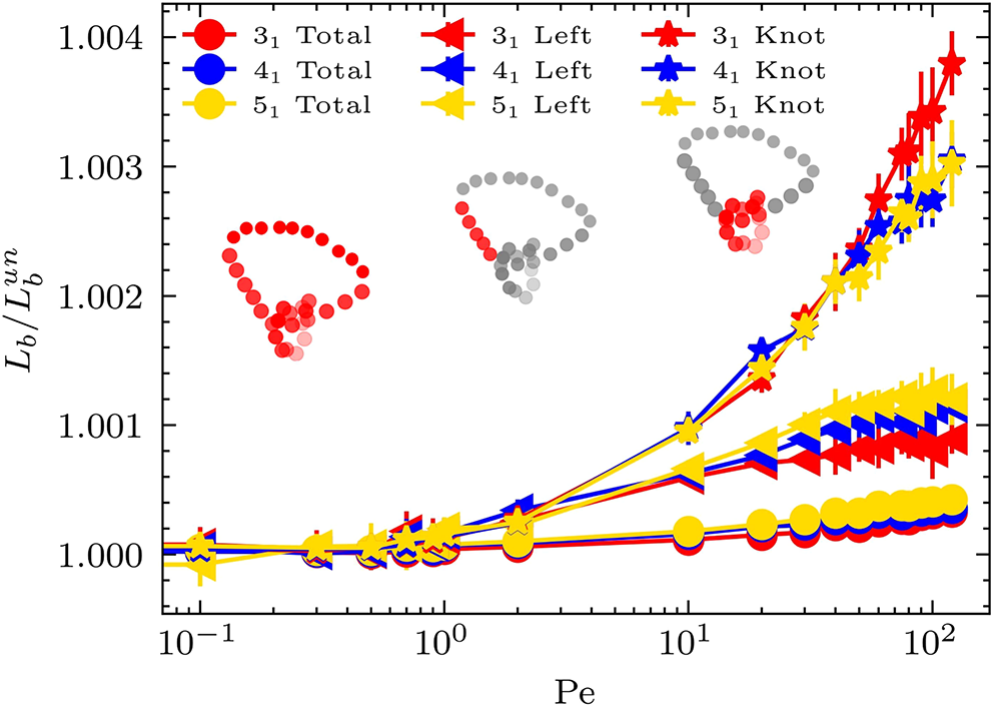
Average bond length of different regions on the ring polymer. Inset diagrams show these regions in red. Circles represent the average bond length of the entire ring. Stars represent the knotted region. The left triangular symbol indicates the region to the left of the knot (defined as the left five beads). Different knot types are presented in different colors.

### Approximate Theory for Activity-induced Knot Tightening

Now, we propose a theory and apply it to explain activity-induced knot tightening based on several approximations. The theory includes three steps: derive (i) the bond stretch versus *Pe*; (ii) the effective stretching force; and (iii) the knot size.

To perform step (i), we have obtained the dependence of the average bond length on *Pe* as *L*_b_ as given in eq 11 in the Method section. In step (ii), we propose that the effective stretching force is proportional to *L*_b_. Accordingly, we have

12 $$f_{s}∼k_{\mathrm{spring}}\sqrt{1+C{\mathrm{Pe}}^{2}}$$

where *k*_spring_ acts as a spring constant and, together with constant *C*, will be determined by the fit to the simulation results. In the final step, we relate the knot size and *f*_*s*_ using the scaling relations derived by Caraglio et al.^49^ Eventually, we have

13 $$L_{k}-L_{k}^{\min}∼{\sqrt{1+C{\mathrm{Pe}}^{2}}}^{-t/0.588}$$

in the large force or large-activity regime; and

14 $$L_{k}-L_{k}^{\min}∼N^{t}\left(1-AN^{0.588}\sqrt{1+C{\mathrm{Pe}}^{2}}\right)$$

in the low-force or low-activity regime.^49^ Here, *L*_*k*_^min^ is the size of the tightest knot, *t* = 0.4 ± 0.1 is a scaling exponent, *N* = 200 is the polymer length, and *A* is a dimensionless coefficient all defined by Caraglio et al. Our fits to the simulation results in Figure 2a yield *t* ≈ 0.44, 0.46, and 0.44 for 3_1_, 4_1_, and 5_1_ knots respectively, in alignment with the value obtained for mechanically stretched polymer knots. These results and analyses suggest that active force indeed induces a stretching effect on the knot.

### Dynamics of Knot Tightening by Activity

Our additional analysis shows that the situation is more complex in terms of the dynamics of knot tightening. To analyze the dynamical evolution of knot localization, we first compare tightening effects across activity levels by running simulations with initial knot size of *L*_*k*_ = 100 and tracking the knot size over time, where time is normalized using the rotational diffusion time, τ_*r*_ = 1/*D*_*r*_. This normalization accounts for the persistence of active force orientation: higher *P*e results in longer memory of the active force’s orientation, amplifying the directed tightening effect. For systems with *Pe* > 30, we observe that knot localization initiates at τ_*r*_ (Figure 4a,b). This tightening phase ends by ∼10^1^τ_*r*_, after which knots remain strongly localized with minimal size fluctuations. However, localization dynamics are not universally monotonic (Figure 4b,c). At high activity (*Pe* = 120), transient twisted polymer bundles form (Figures 1c and 4c). When these bundles coincide with the knotted region, they create entanglements that impede tightening, an effect absent in stretched polymer knots. Note that these entanglements should be distinguished from topological ones (e.g., knots), as they solely induce local density variations and conformational distortions without altering the ring’s topology. This hindrance delays localization by up to ∼10^1^τ_*r*_ (Figure 4b red curve). Figure 4c illustrates these two distinct pathways. The top row shows smooth tightening, where twisted bundles emerge away from the knot, allowing unimpeded tightening of knots. The bottom represents the pathway where bundles overlap with the knot, requiring partial untwisting before localization can proceed.

**Figure 4:**
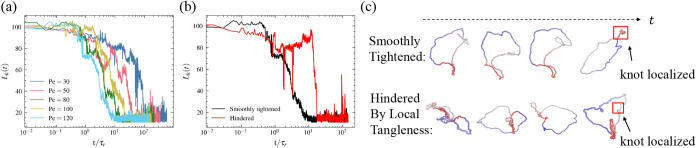
Dynamics of knot tightening. (a) Time evolution of knot size *L*_*k*_(*t*) for polymer knots under varying activity levels. (b) Knot size dynamics under *Pe* = 120. The black curve corresponds to smooth, unimpeded tightening, while the red curve illustrates tightening delayed by temporary local entanglements. (c) Schematic representation of knot tightening pathways in (b). Top: smooth contraction; bottom, entanglement-hindered tightening.

Other than the emergence of the impedance pathway in active knots, we also observe that the distributions of active knot size are significantly different from the knot under pure stretch (Figure 5). To compare the distributions of knot size under these two scenarios, we use the *Pe* – *f*_*s*_ mapping in Figure 2b. Although mean knot sizes are close, the shapes of distributions are distinct. We quantify this shape difference by skewness γ, which measures how skewed a distribution is about its mean, and kurtosis κ, which quantifies the “tail” of the distributions (see SI). In contrast to the stretched knots, the distributions of active knot size are significantly skewed toward the left compared to stretched counterparts (γ_*act*_ > 1 > γ_*stretch*_), indicative of the nonequilibrium nature of the active model (Figure 5a–c). For *Pe* > 10, it can be seen from the insets of Figure 5b,c that the knot size distributions of active knots have a notable long tail extending to the larger knot size, with kurtosis κ_*active*_ ≫ 1 > κ_*stretch*_. When the stretch force *f*_*s*_ gets large, knots become strongly localized and remain tightened throughout the simulation (Figure 5c). However, for large *Pe*, active knots exhibit the breathing effect, where knot size switches back and forth from maximally *L*_*k*_ = 74 to minimally *L*_*k*_ = 11 (Figure 5c,d). Our results suggest that although using our theory one can map activity to effective stretch force when considering time-averaged physical observables in steady state, two systems are dynamically distinct.

**Figure 5:**
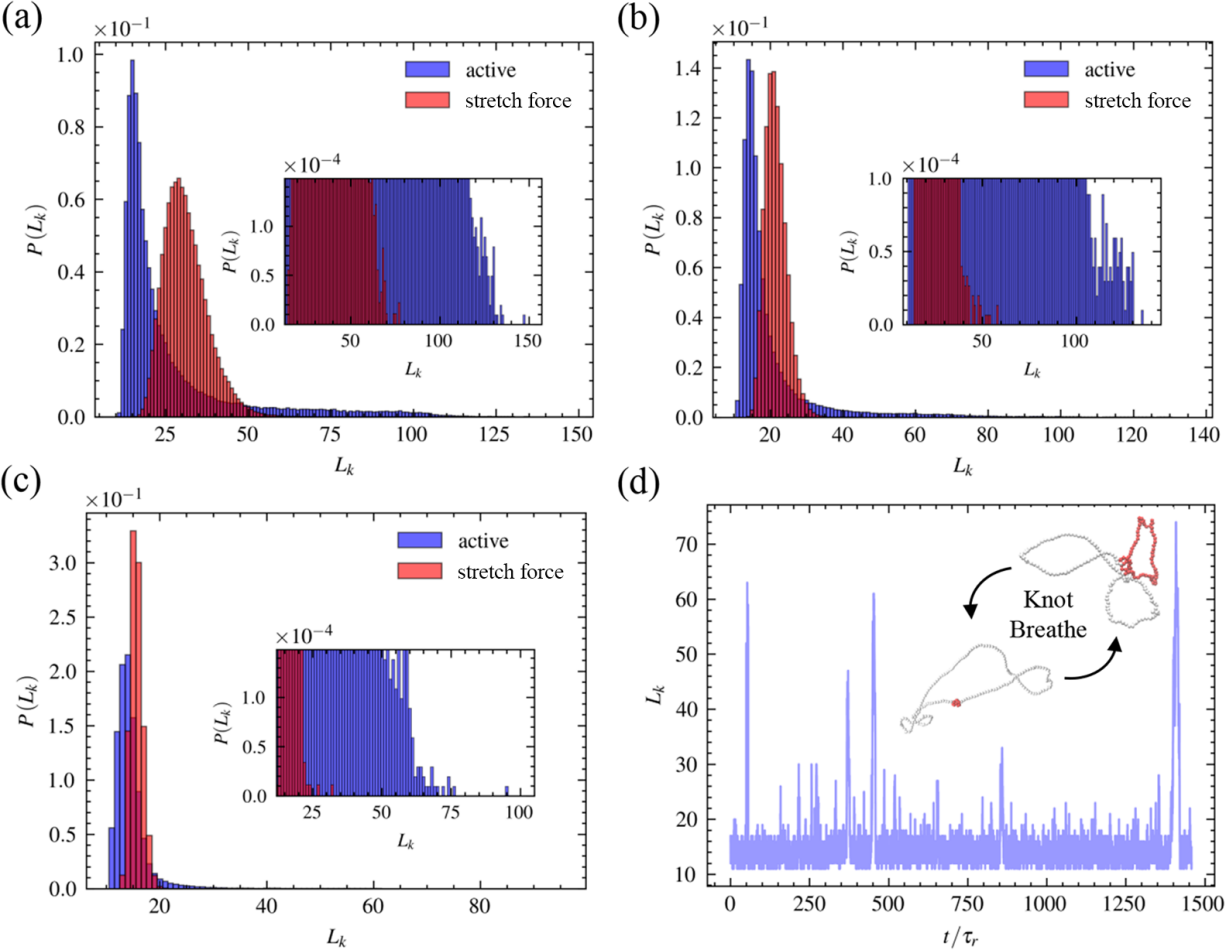
Distributions of active and stretched trefoil knots. (a) *Pe* = 10 and *f*_*s*_ = 1. The skewness γ_*active*_ and kurtosis κ_*active*_ of active knot distribution are γ_*active*_ = 2.36 and κ_*active*_ = 5.39. The skewness γ_*strech*_ and kurtosis κ_*strech*_ of stretched knot distribution are γ_*strech*_ = 0.85 and κ_*strech*_ = 1.11. (b) *Pe* = 20 and *f*_*s*_ = 2. For active knots, skewness and kurtosis are γ_*active*_ = 3.56 and κ_*active*_ = 15.36. For stretched knots, skewness and kurtosis are γ_*strech*_ = 0.79 and κ_*strech*_ = 1.77. (c) *Pe* = 120 and *f*_*s*_ = 5. For active knots, skewness and kurtosis are γ_*active*_ = 5.47 and κ_*active*_ = 47.52. For stretched knots, skewness and kurtosis are γ_*strech*_ = 0.52 and κ_*strech*_ = 0.87. (d) Knot breathing effect in trefoil knots at *Pe* = 120. The inset shows configurations of the maximally large knot of size *L*_*k*_ = 74 and a minimally tightened knot of size *L*_*k*_ = 11. Red colored monomers represent the knot cores.

### Effect of Activity on Conformational Size

Another intriguing phenomenon we observe is the effect of randomly oriented active forces on conformations of knots. Figure 6 shows the time-averaged radius of gyration of polymer conformations, *R*_*g*_, as a function of *Pe*. In addition to the stretching effect, *R*_*g*_ of unknotted polymer rings exhibits a nonmonotonic change with *Pe* (black dashed line in Figure 6a). This shrinking effect is consistent across all lengths (*L* = 20, 50, 100, 300) examined (see SI). The dip of *R*_*g*_ around *Pe* ≈ 2 indicates an activity-induced shrinking effect, observed also in active chains.^8,12^ As activity further increases, polymers expand because monomers tend to move apart while creating additional space that reduces interactions among them. However, when ring polymers are not trivially knotted, the low-activity shrinkage disappears (Figure 6 red, blue, and yellow lines). Instead, *R*_*g*_ of the knotted polymer rings monotonically increases with elevated activity, leading to a $R_{g}∼{\mathrm{Pe}}^{1/3}$ scaling law.^11^

**Figure 6:**
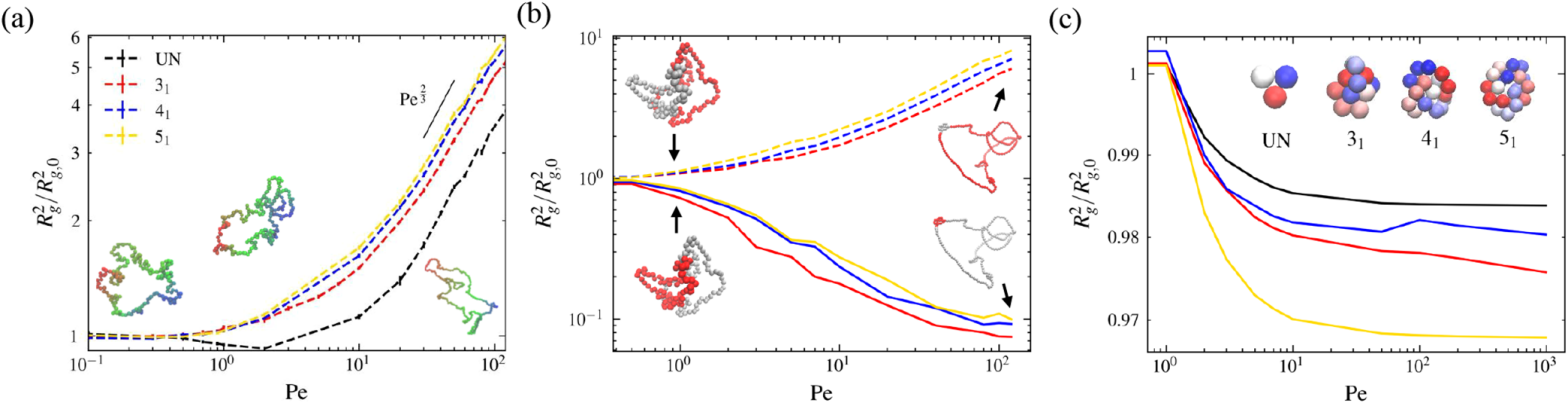
Polymer size under different levels of activity. (a) Normalized radius of gyration *R*_*g*_^2^/*R*_*g*,0_^2^ of knotted and unknotted active ring polymers for *N* = 200. Only unknotted rings show a nonmonotonic change in *R*_*g*_^2^. Inset are, from left to right, conformations of unknotted active rings at equilibrium *Pe* = 0, *Pe* = 2, and *Pe* = 120. For *Pe* ≫ 1, *R*_*g*_^2^ scales with ${\mathrm{Pe}}^{2/3}$ . (b) Normalized radius of gyration of the knotted and unknotted regions for each knot type of length *N* = 200. Broken lines represent the unknotted regions. Solid lines represent the knotted regions. Inset shows polymer knots at *Pe* = 0 (left) and *Pe* = 120 (right). Conformations at the top and the bottom represent the same polymer knots with unknotted and knotted regions colored in red. (c) Normalized radius of gyration for different small knots. Insets are conformations of knots used to measure *R*_*g*_. For trivial ring polymer, we use length *N* = 3. For 3_1_, 4_1_, and 5_1_ knots, we use *N* = 16, 21, 22, respectively.

To clarify the absence of shrinkage in knotted active rings, we separately analyze the average radius of gyration (*R*_*g*_) of the knotted and unknotted regions (Figure 6b). We find that, as the knot tightens, the average radius of gyration of the knotted region (*R*_*g*,knot_) decreases, while that of the unknotted region (*R*_*g*,unknot_) increases, consistent with expectations. The monotonic decrease in *R*_*g*,knot_ and corresponding increase in *R*_*g*,unknot_ across all activity levels indicates that the activity-induced shrinkage at low *Pe* is effectively counterbalanced by the tightening of the knot, which frees up space for the expansion of unknotted segments.

To eliminate the influence of segment reallocation between knotted and unknotted regions, we performed additional simulations on short knotted polymers (*N* = 16, 21, and 22 for the 3_1_, 4_1_, and 5_1_ knots, respectively), as shown in Figure 6c. Surprisingly, we find that activity consistently leads to a reduction in polymer conformational size (we note that the situation is a bit more complex. See SI for details). For comparison, we also include results for a minimal unknotted ring (*N* = 3), which shows a similar shrinking trend. Notably, the knotted rings exhibit a more pronounced shrinkage than the unknot. These findings indicate that activity induces effects beyond simple stretching, suggesting a more complex interplay between activity and polymer topology.

Different results of long and short active knotted polymers in Figure 6a,6c can be understood in the following way. The *R*_*g*_-Pe curve results from the superposition of two competing effects: activity-induced shrinkage, which tends to reduce *R*_*g*_, and reallocation of segments from knotted to unknotted portions, which tends to increase *R*_*g*_. In short active polymers, the absence of an unknotted portion eliminates the second effect, leading to a decrease of *R*_*g*_ with increasing *Pe*. In long active knotted polymers, however, segment reallocation can dominate, causing *R*_*g*_ to increase with activity.

The fact that shrinkage occurs in short and compacted active knots suggests that the situation may be analogous to motility-induced phase separation (MIPS) in active Brownian particle systems,^8,12,19^ where active forces drive particles into dense, dynamically arrested clusters. In the context of knotted polymers, active forces may promote local jamming of monomers, resulting in a metastable, compact state. This effect can also be seen in the conformational snapshots shown in Figure 1c, where stiffened segments form twisted bundles, indicative of a coexistence between stretching and particles’ slowdown mediated by activity.

The distinct behaviors observed between unknotted and knotted polymers, as well as among different knot types in Figure 6c, indicate that the spatial arrangement of beads imposed by the knot type can significantly influence the collective action of active forces on the beads. These findings show that knotting impacts active polymers not only through stretching, but also by altering their spatial organization.

## Conclusions

In summary, we uncover a range of nontrivial phenomena emerging from the interplay between active forces, chain connectivity, and topological constraints. The combination of activity and connectivity gives rise to an effective stretching effect, as evidenced by both bond extension and knot tightening. We develop a theoretical framework that links activity to an effective stretching force, offering a mechanistic explanation for activity-induced knot localization. Beyond this stretching effect, activity manifests more complex effects in dynamical and conformational aspects, where some effects are even seemingly contradictory to stretching. Dynamically, the distribution of active knot size is distinct from the passive knots under stretch, showcasing its nonequilibrium nature. Also, knot breathing exists even at high activity level, while the equilibrium counterpart is strong localized throughout the simulation. Conformation-wise, activity can also lead to conformational compaction, particularly in small polymer rings, regardless of whether they are knotted or unknotted. In larger rings, however, the influence of activity on polymer conformation becomes more nuanced, as multiple mechanisms–such as stretching, segment redistribution between knotted and unknotted regions, and the compaction observed in short rings–may act simultaneously.

Intriguingly, activity can shrink very short chain conformations, and knotting appears to enhance this activity-induced shrinkage. This enhancement may be caused by the fact that knotting alters the spatial organization of monomers, especially by inducing crossing and entanglement, which affects the collective behavior of active forces on monomers. Future studies can further uncover the mechanism of enhanced shrinkage.

These findings may have significant biological implications, particularly in the context of DNA and other biopolymers where both knotting and activity are prevalent.^12,40,41^ Understanding how activity and topology influence polymer structure could inform models of chromosomal organization, transcription dynamics, and the behavior of synthetic active materials.

## Supplementary Material













## Notes

Simulation methods and parameters; properties of active unknotted and knotted rings; analysis of bond and angle distributions in ideal active polymers; dynamics and localization of knots; coordinate transformation and analytical derivation of bond length and radius of gyration; supplementary videos showing knot behavior at various Peclét numbers (PDF)
Movie S1 shows a trefoil knot at *Pe* = 0 (MP4)
Movie S2 shows a trefoil knot at *Pe* = 20 (MP4)
Movie S3 shows a trefoil knot at *Pe* = 120 (MP4)
Movie S4 *Pe* = 120 knot shrinks smoothly (MP4)
Movie S5 *Pe* = 120 knot shrinks but hindered (MP4)
