# Supplementary material for: Behavior of Active Polymer Knots
Source: Macromolecules. 2025 Oct 7;58(20):11229–36. doi: 10.1021/acs.macromol.5c01381 (PMC12772128; doi:10.1021/acs.macromol.5c01381)
Supplement: Supplementary file 1 [file ma5c01381_si_001.pdf]

Supporting information:
Behavior of active polymer knots

Zhiyu Zhang[1], Longfei Li[2], Yongjian Zhu[1], Rui Zhang[3], Mingcheng Yang[2], and Liang Dai*[1]

[1]Department of Physics, City University of Hong Kong, Hong Kong, 999077, China
[2]Beijing National Laboratory for Condensed Matter Physics and Laboratory of Soft Matter Physics, Institute of Physics, Chinese Academy of Sciences, Beijing, 100190, China
[3]Department of Physics, Hong Kong University of Science and Technology, Hong Kong, 999077, China
**Email* : *liangdai@cityu.edu.hk*

Contents

1 Simulation Method **2**

2 Properties of Active Brownian Ring **3**

3 Properties of Small Active Brownian Knots **5**

4 Analysis of Bond and Angle in Ideal Active Brownian Polymer Rings **6**

5 Dynamics of Knots on The Active Brownian Ring Polymer **7**

6 Coordinate Transformation of Coupled Dynamical Equation **8**

7 Averaged Bond Length L_b and Radius of Gyration $\mathrm{R_g}$ of Ideal Topologically-Unconstrained Active Ring Polymer **10**

8 Supplementary Movies **12**

1 Simulation Method

The model that we use in this paper is a simple flexible and knotted polymer ring, which is a collection of monomer beads of diameter σ that each undergoes Brownian motion and is acted on by a self-propelling active force $\mathbf{f}_{\text{act}}$ in the direction of its orientation. We perform active Brownian dynamics simulation using HOOMD-blue[1]. The active part in the simulation comes in as an active force applied, which is set to be constant in magnitude and diffuses rotationally with the orientation of each bead[2, 3]. The beads are self-excluding, enforced by Weeks-Chandler-Andersen (WCA) potential, and connected via spring forces. For bond energy, we apply the Kremer-Grest model where we add a finitely extensible nonlinear elastic (FENE) potential to lock the topology of the knotted polymer ring[4, 5]. The resulting equation of motion for each bead in three dimensions reads as

$$\gamma \frac{\mathrm{d}\mathbf{r}_{\mathrm{i}}(\mathrm{t})}{\mathrm{dt}} = -\sum_{j} \nabla \mathrm{V}_{\mathrm{tot}}(\mathbf{r_i} - \mathbf{r_j}) + \mathbf{f}_{\mathrm{i}}^{\mathrm{r}}(\mathrm{t}) + \mathbf{f}_{\mathrm{act},i}, \quad (1)$$

where γ is the viscous drag coefficient, $\mathbf{f}_{\mathrm{i}}^{\mathrm{r}}$ is the Gaussian random force that averages zero and follows $\langle \mathrm{f}_{\alpha,\mathrm{i}}^{\mathrm{r}}(\mathrm{t}), \mathrm{f}_{\beta,\mathrm{j}}^{\mathrm{r}}(\mathrm{t}') \rangle = 2\mathrm{k_B T}\gamma\delta_{ij}\delta_{\alpha\beta}\delta(t-t')$. The last term in the equation of motion is the active force $\mathbf{f}_{\mathrm{act}} = \mathrm{f_a}\hat{\mathbf{u}}_{\mathrm{i}}$, where $\hat{\mathbf{u}}_{\mathrm{i}}$ is the orientation of the particle which undergoes rotational diffusion

$$\gamma_r \frac{\mathrm{d}\hat{\mathbf{u}}_{\mathrm{i}}(\mathrm{t})}{\mathrm{dt}} = \hat{\mathbf{u}}_{\mathrm{i}} \times \mathbf{\Lambda}_{\mathrm{i}}^{\mathrm{r}}, \quad (2)$$

with $\langle \Lambda_{\alpha,\mathrm{i}}^{\mathrm{r}}(\mathrm{t}), \Lambda_{\beta,\mathrm{j}}^{\mathrm{r}}(\mathrm{t}') \rangle = 2\mathrm{k_B T}\Lambda\gamma\delta_{ij}\delta_{\alpha\beta}\delta(t-t')$. The net potential $\mathrm{V_{tot}} = \mathrm{V_{FENE}} + \mathrm{V_{excl}}$ has two parts, where the first part is from FENE potential $\mathrm{V_{FENE}}$

$$\mathrm{V_{FENE}} = -15\epsilon \frac{\mathrm{r}_0^2}{\sigma^2} \ln \left(1 - \left(\frac{\mathrm{r}}{\mathrm{r}_0}\right)^2\right)$$

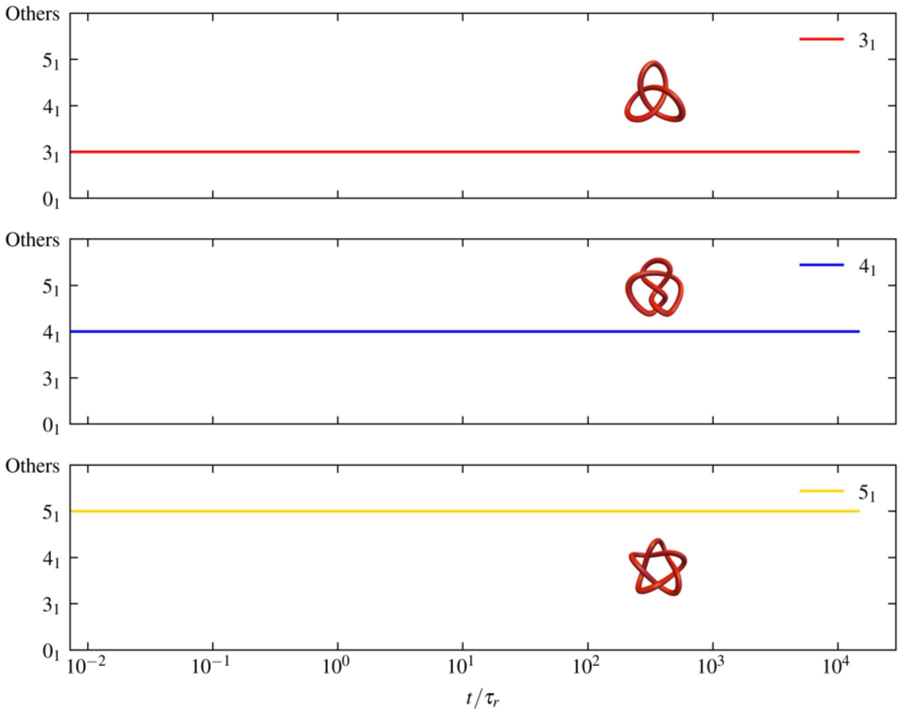

Figure 1: Knot type as a function of simulation. The results are used to confirm that no segment-segment crossing occurs in the simulations.

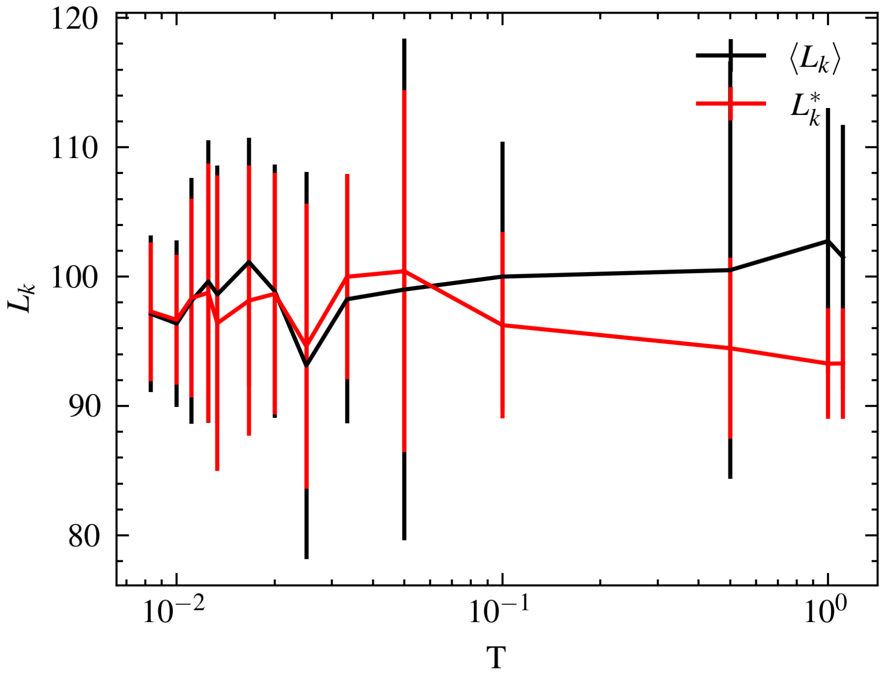

Figure 2: Knot size versus ambient temperature. There is no apparent change in knot size as temperature decreases as predicted in theory when ϵ is fixed when changing temperature.

with $\mathrm{r}_0 = 1.5\sigma$ and $\epsilon = 1$. $\mathrm{V}_{\mathrm{excl}}$ is the WCA exclusion potential among non-neighboring beads

$$\mathrm{V}_{\mathrm{excl}} = \begin{cases} 4\epsilon\left[\left(\frac{\sigma}{\mathrm{r}}\right)^{12} - \left(\frac{\sigma}{\mathrm{r}}\right)^{6}\right] + \epsilon & \mathrm{r} < 2^{\frac{1}{6}}\sigma \\ 0 & \text{otherwise.} \end{cases} \tag{3}$$

We run simulations for $10^3 - 10^5 \tau_r$. We provide several movies to better see the active rings in real-time at different activity levels (see supplementary movies). We determine the activity with the Péclet number $\mathrm{Pe} \equiv \mathrm{f}_{\mathrm{act}}\sigma/k_B T$, where $k_B T$ is the thermal energy of the surrounding environment. Other than the shrinking effective radius of the bead concurred with the elevated active force as mentioned in the main text, we increase the system's activity, i.e. value of Pe, by cooling the ambient temperature while holding $\mathrm{f}_{\mathrm{act}}\sigma \equiv 1$ to facilitate simulation to ensure that the bond force is sufficient enough to hold beads in place and not over-stretched when Pe is high. Topologies are fixed during the simulation by the KG potential (Fig. 1).

One may think that knot shrinking is caused by lowering temperature[6, 7]. However, we can exclude this possibility, because our simulation system is a flexible ring of hard-spheres (without attraction), different from the chains with attractions in previous studies[6, 7]. It is well known that hard-sphere systems are athermal, i.e. independent of temperature, because there are only two possible energies: $+\infty$ (when overlapped) and 0. Our additional simulations confirmed that our polymer knot sizes with WCA potentials, nearly hard-sphere replusion[8] are insensitive to temperature (Fig. 2). Hence, knot shrinking should be caused by an active force.

2 Properties of Active Brownian Ring

Similar to active Brownian polymer open chain, the unknotted ring polymers exhibit low-activity shrinkage across different system sizes (Fig. 3). The onset of low-activity shrinkage can be more

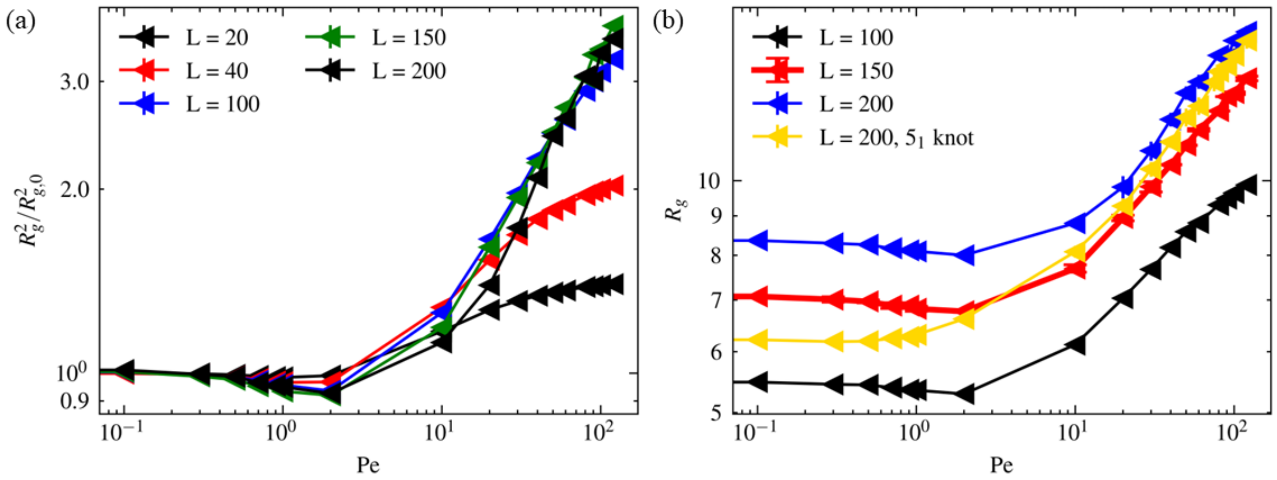

Figure 3: Radius of gyration of rings with the trivial topology. (a)Normalized radius of gyration of polymer with different sizes. For all system sizes examined, R_g decreases at the low activity regime. (b)The radius of gyration of unknotted rings and 5_1 knotted rings.

clearly seen when compared to the knotted ring with similar R_g. A clear monotonic trend is observed for R_g of the 5_1 knotted ring, while active unknotted rings with similar size shrink at low activity (Fig. 3b). This also indicates that the absence of low-activity shrinkage is completely independent of system size and thus not an artifact of reduced size incurred by knots.

For large systems, we have performed simulations for systems with N = 300. Larger systems are not considered as relaxation time becomes too long such that it is computationally cost-expensive. The result for the change of knot size and R_g is shown in Fig. 4.

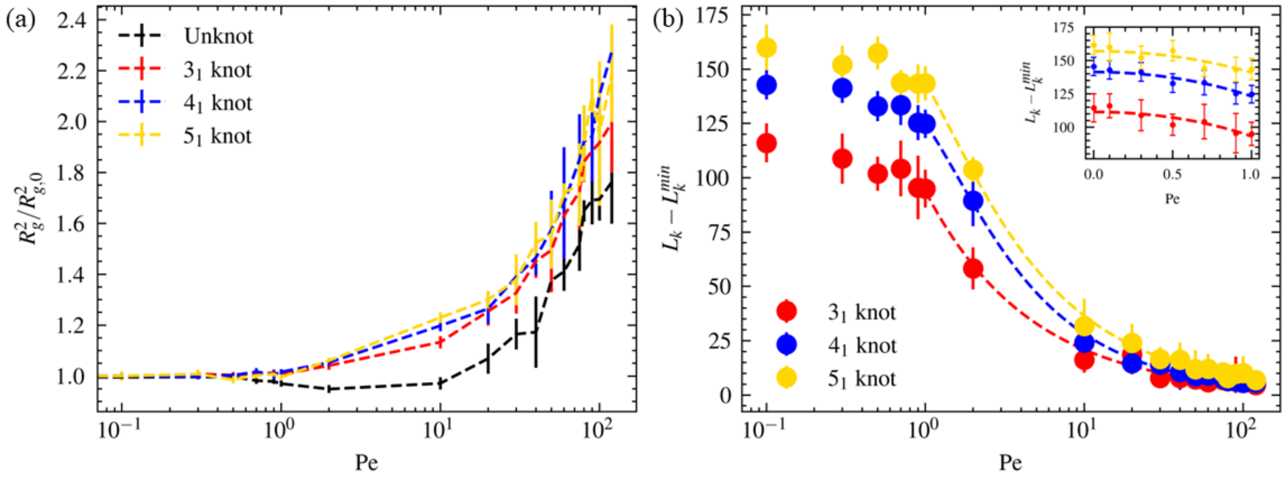

Figure 4: Properties of knotted active polymers of size $N = 300$. (a)The radius of gyration versus Pe. (b)Knot size versus Pe. The average knot size is larger than the system of N = 200. The scaling constant $t = 0.38$, 0.44, and 0.40, respectively.

Other than increased average knot sizes, the localization follows similar decaying behavior, where scaling constant $t = 0.4 \pm 0.1$[9]. The low-activity shrinkage of polymer is quenched in the active rings with nontrivial topology. However, for extremely small rings with trefoil knots, the low activity

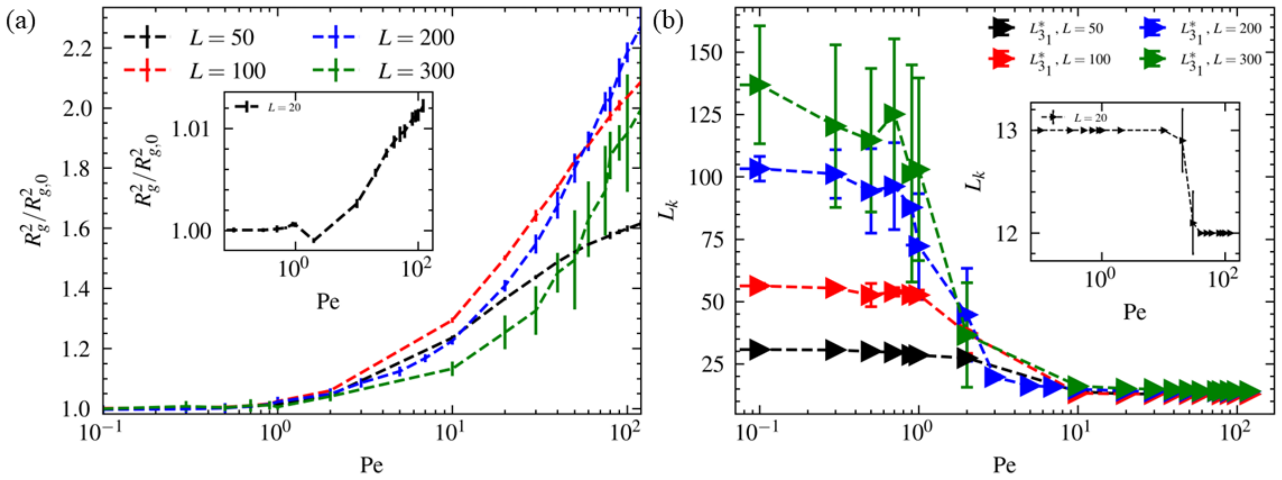

Figure 5: (a)The radius of gyration of rings of different sizes with the trivial topology under different activity. (b)The knot size of trefoil knotted active polymer rings versus Pe.

shrinkage is recovered due to the inability of knots to expand (Fig. 5).

3 Properties of Small Active Brownian Knots

Aside from shrinkage mentioned in the main text, we also see unusual responses of knotted active rings as lengths change.

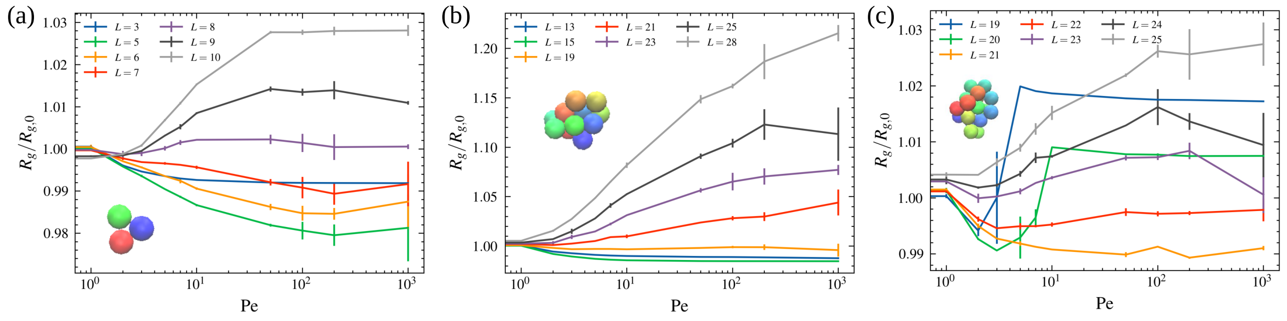

Figure 6: Non-monotonic polymer size change under different activity levels for different topologies. (a) Unknotted ring (b) 3_1 knot (c) 4_1 knot

As shown in Fig. 6, unknotted rings transition from monotonic shrinkage to non-monotonic shrinkage-expansion behavior as the number of monomers increases (Fig. 6a). For the 3_1 knot, increasing the monomer shows a monotonic shrinkage to monotonic expansion phenomenon (Fig. 6b). In contrast, 4_1 knots exhibit distinct behavior in response to monomer number. For small 4_1 knots ($N = 19$ and $N = 20$), the rings initially shrink but undergo sharp expansion at low activity levels, and their sizes abruptly plateau at Pe $\sim 10^1$ (Fig. 6c). With increasing monomer count, 4_1 knots adopt monotonic shrinkage, similar to the unknotted and 3_1 rings. However, further increases in monomer number introduce complexity: at $N = 23$, a non-monotonic regime emerges, characterized by shrinkage at low activity followed by expansion at high Pe. This behavior persists only within a narrow range of monomer numbers, disappearing entirely at $N = 25$—just two monomers beyond its initial appearance. These results demonstrate that the response of knots to activity and chain length

involves diverse phenomena, necessitating further research to fully elucidate how activity drives such complex behaviors.

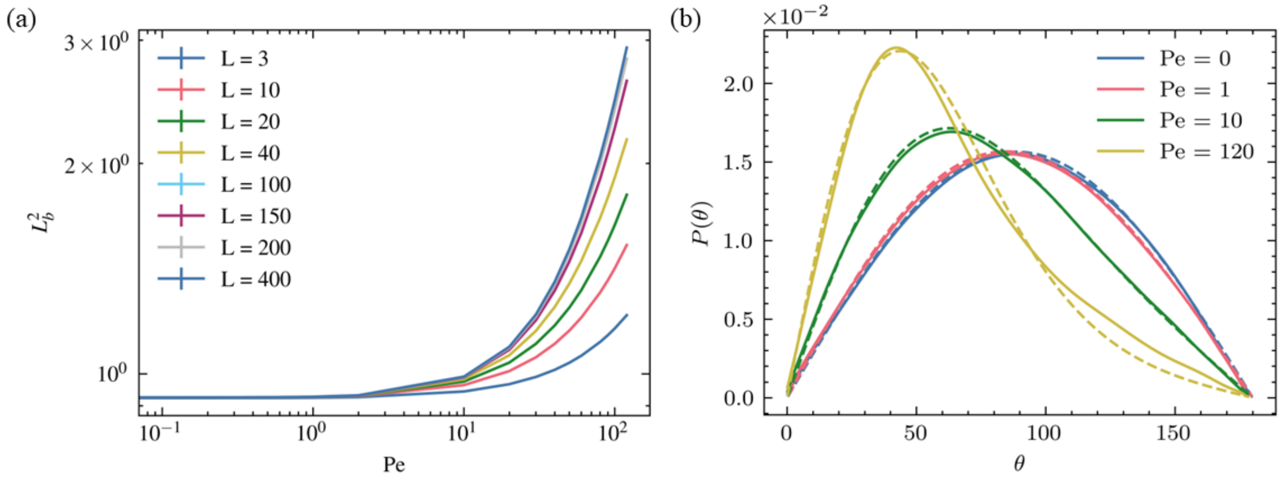

Figure 7: (a)The bond length of ideal active Brownian ring polymer of different lengths at different Pe's. (b)Angular probability distribution of ideal chain polymer $L = 3$ at different activity levels. Dashed lines are fitting according to eqn. 4

4 Analysis of Bond and Angle in Ideal Active Brownian Polymer Rings

In the main text, we presented results for ideal active three-bead chains. Here, we present the data for different lengths (Fig. 7). In the simulation for these polymers, we can change the active force $\mathrm{f_a}$ directly since there is no steric effect among beads (Fig. 8). To remain consistent with the main text, we change k_bT and the result is shown in Fig. 7a.

For rings of L < 200, larger rings will have a more elevated bond stretch. This trend ceases to exist when L reaches 200 beads. This indicates that the average bond in an active Brownian ring depends on the entire ring (this can also be seen analytically in Section 6).

For a force-free three-bead system in equilibrium, the angle between the two bonds follows the probability density function (pdf) $P(\theta) \sim \sin\theta$ since there is no correlation between the two bonds (Fig. 7b blue curve). Consider a force $\mathbf{f}_s$ is applied to the ends of the system. Since probability distribution of bending angle $P(\theta)$ (Fig. 2C inset diagram in the main text) is proportional to $\int e^{-\beta E} \sin\theta r^2 d\theta d\phi dr$ and $E = -f_s L_b \cos\theta$, it follows that, by symmetry ϕ integrates out and r is fixed. That is, a stretched polymer, the angular probability density function (PDF) becomes

$$P_{stretched}(\theta) \sim e^{f_s L_b \cos\theta} \sin\theta, \tag{4}$$

where L_b is the bond length and f_s is the magnitude of stretch force. For the force-free scenario $f_s = 0$, we recover the previous pdf $P(\theta) \sim \sin\theta$. We use eqn.(4) to fit the angular distribution of the ideal three-bead active Brown chain polymer and obtain the effective stretch force, as shown in the main text. Although this approximately maps the active system to the stretched equilibrium considering the most probable angles are similar, at Pe = 120, the angular distribution exhibits a noticeable discrepancy (Fig. 7b yellow curve). After the crossover at $\theta \sim \frac{\pi}{2}$, bond angles in stretched polymer distributed considerably less for $\theta > \frac{\pi}{2}$. This implies that activity is less effective in constraining the bond angles compared to mechanical force.

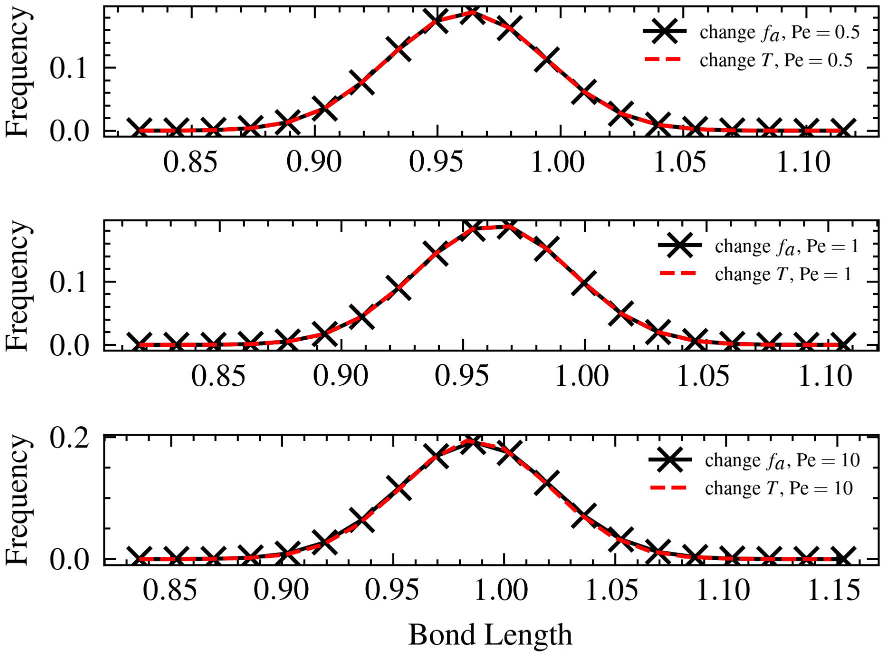

Figure 8: Distribution of bond lengths by changing $\mathrm{f_a}$ versus changing k_BT in active ideal polymer. Top panel: bond length distribution for Pe = 0.5. Middle panel: bond length distribution for Pe = 1. Bottom panel: bond length distribution for Pe = 10.

5 Dynamics of Knots on The Active Brownian Ring Polymer

In the main text, we used notions of skewness γ and kurtosis κ to describe the shape of distributions of knot sizes. Here we give the precise definitions and show some example distributions to give an intuitive picture of these two quantities.

To measure how skewed a distribution is, we use the definition of *sample skewness*

$$\gamma := \frac{m_3}{m_2^{\frac{3}{2}}}, \tag{5}$$

where

$$m_i := \frac{1}{N}\sum_{n=1}^{N}(x_n - \mu)^i, \tag{6}$$

is the ith central moment and μ is the mean.

To quantify the tailedness of a distribution, we use the Fisher's definition such that, for normally distributed samples, the value is 0. The skewness κ is given by

$$\kappa := \frac{m_4}{m_2^2} - 3. \tag{7}$$

To give some examples, normal distribution has approximately zero skewness and kurtosis. Uniform distribution is also not skewed, but on the other hand is platykurtic (negative kurtosis), represented by a thin tail. For a right/left-skewed exponential distribution, the skewness γ is greater/less than 0, and both have kurtosis κ greater than 0. This is shown in Fig. 9.

In addition to what we presented in the main text, we also analyze the locomotion of different knots on the active Brownian ring polymer. Here, we plot in Fig. 10 the Mean-Squared-Displacement

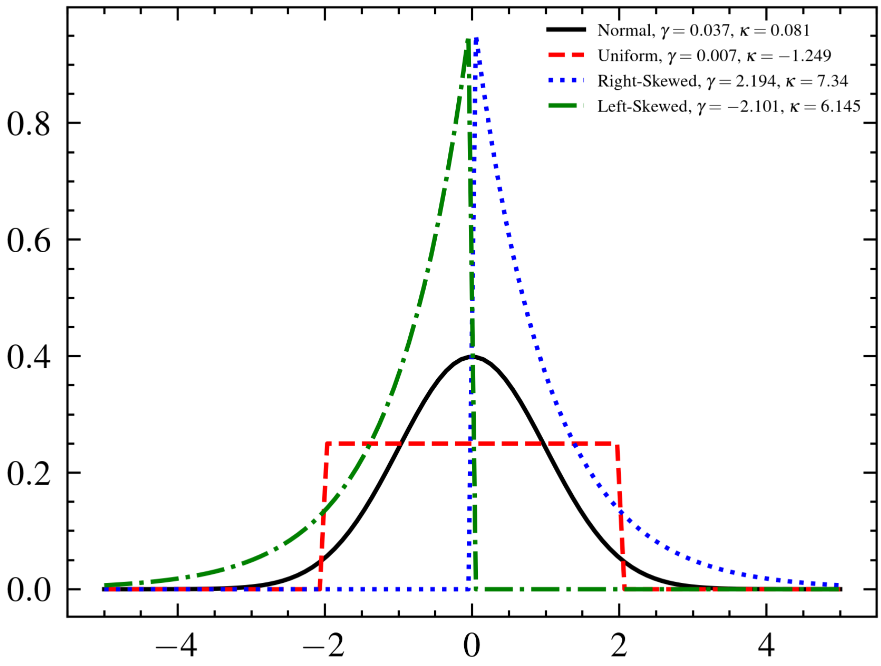

Figure 9: Skewness and kurtosis of different distributions.

(MSD) function of the 3_1 knot. We find that knots diffuse akin to a Brownian walk, which is how knots diffuse on a stretched polymer chain. We also tracked the trajectory of the center of the knots over time (Fig. 10b). δn is the number of beads a knot traveled along the polymer backbone. For all three trials, both forward and backward motions have been observed.

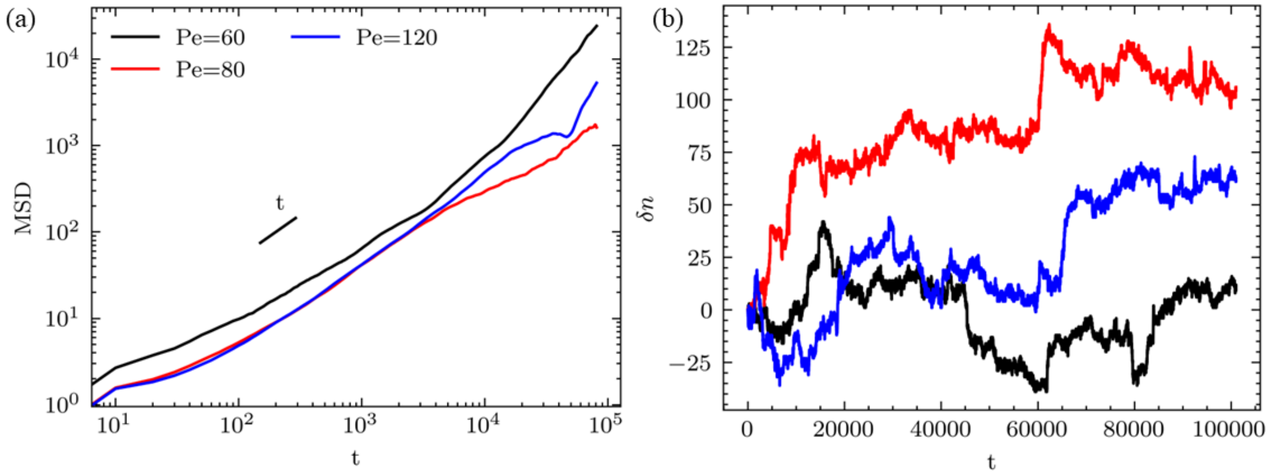

Figure 10: (a)Mean Squared Displacement function versus time of 3_1 knots on active polymer of size N = 200. (b)Drift of trefoil knots along the polymer backbone. There is no unidirectional translocation of knots.

6 Coordinate Transformation of Coupled Dynamical Equation

To analyze the conformational properties of the active Brownian rings and map the activity to an effective stretch, we show that by using coordinates transformation one can transform the second-order

stochastic differential equation (SDE) into an Orstein-Ulenbeck-type SDE. The following derivation follows from the well-known text by Doi and Edwards[10]. We consider the following dynamical equation of motion:

$$\gamma \frac{\mathrm{d}\mathbf{r}_{\mathrm{i}}(\mathrm{t})}{\mathrm{dt}} = k\frac{\partial^2 \mathbf{r}_i}{\partial i^2} + \mathbf{f}_{\mathrm{i}}^{\mathrm{r}}(\mathrm{t}) + \mathbf{f}_{\mathrm{act,i}}, \tag{8}$$

a continuum spring-bead model, with

$$\begin{aligned} \mathbf{r}_0 &= \mathbf{r}_N \\ \mathbf{r}_{N+1} &= \mathbf{r}_1 \end{aligned} \tag{9}$$

as the conditions for ring closure. First, we write our coordinate transformation of $\frac{\mathrm{d}\mathbf{r}_{\mathrm{i}}(\mathrm{t})}{\mathrm{dt}}$ as

$$\mathbf{X}_{\mathrm{p}}(\mathrm{t}) = \int_0^N \phi_{pi}\mathbf{r}_{\mathrm{i}}(\mathrm{t})\, di, \tag{10}$$

where ϕ_{pn} satisfies the following condition

$$\gamma_p \frac{\partial \mathbf{X}_{\mathrm{p}}(\mathrm{t})}{\partial t} = -k_{\mathrm{p}}\mathbf{X}_{\mathrm{p}}(\mathrm{t}) + \tilde{\mathbf{f}}_{\mathrm{p}}^{\mathrm{r}}(\mathrm{t}) + \tilde{\mathbf{f}}_{\mathrm{act},p}, \tag{11}$$

where γ_p, we will shortly see, is a constant to facilitate coordinate transformation. Multiply each side of eqn.(8) by ϕ_{pn} and integrate with i from 0 to N we obtain

$$\gamma_p \frac{\partial \mathbf{X}_{\mathrm{p}}(\mathrm{t})}{\partial t} = \int_0^N \phi_{pi}\frac{\partial \mathbf{r}_{\mathrm{i}}(\mathrm{t})}{\partial t}\, di = \frac{\gamma_{\mathrm{p}}}{\gamma}\int_0^N \phi_{pi}\Big(k\frac{\partial^2 \mathbf{r}_i}{\partial i^2} + \mathbf{f}_{\mathrm{i}}^{\mathrm{r}}(\mathrm{t}) + \mathbf{f}_{\mathrm{act,i}}\Big). \tag{12}$$

Using integration by parts twice, we can write RHS as

$$\begin{aligned} RHS = \frac{\gamma_{\mathrm{p}}}{\gamma}\left[k\phi_{pi}\frac{\partial \mathbf{r}_{\mathrm{i}}}{\partial i}\right]_0^N - \frac{\gamma_{\mathrm{p}}}{\gamma}\left[k\frac{\partial \phi_{pi}}{\partial i}\mathbf{r}_{\mathrm{i}}\right]_0^N \\ + \frac{\gamma_{\mathrm{p}}}{\gamma}\int_0^N \left[k\frac{\partial^2 \phi_{pi}}{\partial i^2}\mathbf{r}_{\mathrm{i}} + \phi_{pi}\Big(\mathbf{f}_{\mathrm{i}}^{\mathrm{r}}(\mathrm{t}) + \mathbf{f}_{\mathrm{act,i}}(t)\Big)\right]. \end{aligned} \tag{13}$$

This result should be equivalent to the RHS of eqn. (11). This holds if we allow the first two terms to be 0, terms in the integral to be equal, and lastly

$$\begin{aligned} \tilde{\mathbf{f}}_{\mathrm{p}}^{\mathrm{r}}(\mathrm{t}) &= \frac{\gamma_{\mathrm{p}}}{\gamma}\int_0^N \phi_{pi}\mathbf{f}_{\mathbf{i}}\, di, \\ \tilde{\mathbf{f}}_{\mathrm{act},p} &= \frac{\gamma_{\mathrm{p}}}{\gamma}\int_0^N \phi_{pi}\tilde{\mathbf{f}}_{\mathrm{act,i}}\, di. \end{aligned} \tag{14}$$

Equating the terms in the integral to the term from eqn. (13) and eqn. (11)gives

$$\frac{k\gamma_{\mathrm{p}}}{\gamma}\frac{\partial^2 \phi_{pi}}{\partial i^2} = -k_p\phi_{pi}, \tag{15}$$

Without ring closure condition (9), the first term is 0, as explained in Doi and Edwards' text. Since we are dealing with rings, the first term is not 0. Instead, (9) gives a condition on ϕ_{pn}:

$$\begin{aligned} \frac{\gamma_{\mathrm{p}}}{\gamma}\left[k\phi_{pi}\frac{\partial \mathbf{r}_{\mathrm{i}}}{\partial i}\right]_0^N &= \frac{k\gamma_{\mathrm{p}}}{\gamma}\left(\phi_{pN}\frac{\partial \mathbf{r}_{\mathrm{i}}}{\partial i}\bigg|_{n=N} - \phi_{p0}\frac{\partial \mathbf{r}_{\mathrm{i}}}{\partial i}\bigg|_{n=0}\right) \\ &= \frac{k\gamma_{\mathrm{p}}}{\gamma}\left(\phi_{pN}(\mathbf{r}_1 - \mathbf{r}_N) - \phi_{p0}(\mathbf{r}_1 - \mathbf{r}_N)\right). \end{aligned} \tag{16}$$

Hence, the first two terms of eqn. (13) gives

$$\begin{aligned} &\frac{\gamma_\mathrm{p}}{\gamma}\left[k\phi_{pi}\frac{\partial \mathbf{r}_\mathrm{i}}{\partial i}\right]_0^N - \frac{\gamma_\mathrm{p}}{\gamma}\left[k\frac{\partial \phi_{pi}}{\partial i}\mathbf{r}_\mathrm{i}\right]_0^N \\ &\quad = \frac{k\gamma_\mathrm{p}}{\gamma}\Bigg[\phi_{pN}(\mathbf{r}_1-\mathbf{r}_N) - \phi_{p0}(\mathbf{r}_1-\mathbf{r}_N)\Bigg] - \frac{k\gamma_\mathrm{p}}{\gamma}\left(\frac{\partial \phi_{pi}}{\partial i}\bigg|_{n=N}\mathbf{r}_N - \frac{\partial \phi_{pi}}{\partial i}\bigg|_{n=0}\mathbf{r}_0\right) \\ &\quad = \frac{k\gamma_\mathrm{p}}{\gamma}\left\{\Big(\phi_{pN}-\phi_{p0}\Big)\mathbf{r}_1 - \left[\Big(\phi_{pN}-\phi_{p0}\Big) - \frac{\partial \phi_{pi}}{\partial i}\bigg|_{n=N} + \frac{\partial \phi_{pi}}{\partial i}\bigg|_{n=0}\right]\mathbf{r}_N\right\} = 0 \end{aligned} \tag{17}$$

For the above to be true, we must have

$$\begin{aligned} \phi_{pN} &= \phi_{p0} \\ \frac{\partial \phi_{pi}}{\partial i}\bigg|_{n=N} &= \frac{\partial \phi_{pi}}{\partial i}\bigg|_{n=0} \end{aligned} \tag{18}$$

Now, it is easy to see that from the conditions that we derived for ϕ_{pi} (eqn. (15) and eqn. (18)) we obtain

$$\begin{aligned} \phi_{pi} &= \frac{1}{N}\cos\left(\frac{p\pi i}{N}\right) \qquad (p \in even) \\ k_p &= \frac{k\gamma_\mathrm{p}}{\gamma}\left(\frac{p\pi}{N}\right)^2, \end{aligned} \tag{19}$$

and γ_p can be chosen arbitrarily. We choose $\gamma_p = 2N\gamma$ following previous literature Here, after imposing ring closure on the open linear Rouse chain model, only *odd* normal modes survive. Lastly, the backward transformation gives

$$\mathbf{r}_\mathrm{i}(\mathrm{t}) = 2\sum_{p=1}^{\infty}\phi_{pi}\mathbf{X}_\mathrm{p}(\mathrm{t}). \qquad (p \in even) \tag{20}$$

7 Averaged Bond Length L_b and Radius of Gyration $\mathrm{R_g}$ of Ideal Topologically-Unconstrained Active Ring Polymer

For our model, we can consider the dynamics (eqn. (1) and (2)) in a continuum limit and preserve the simple harmonic potential. Note that this model is similar to the active Rouse model, where beads are isotropic (no rotational diffusion). In the continuum limit, we have

$$\gamma\frac{\mathrm{d}\mathbf{r}_\mathrm{i}(\mathrm{t})}{\mathrm{dt}} = -k\frac{\partial^2 \mathbf{r}_i}{\partial i^2} + \mathbf{f}_\mathrm{i}^\mathrm{r}(\mathrm{t}) + \mathbf{f}_\mathrm{i}^\mathrm{act}, \tag{21}$$

and

$$\gamma_r\frac{\mathrm{d}\hat{\mathbf{u}}_\mathrm{i}(\mathrm{t})}{\mathrm{dt}} = \hat{\mathbf{u}}_\mathrm{i} \times \mathbf{\Lambda}_\mathrm{i}^\mathrm{r}. \tag{22}$$

Using the coordinate transformation eqn. (10) and eqn. (19),

$$\mathbf{X}_\mathrm{p}(\mathrm{t}) = \int_0^N \frac{1}{N}\cos\left(\frac{\mathrm{ip}\pi}{N}\right)\mathbf{r}_\mathrm{i}(\mathrm{t})\,di \qquad (p \in even) \tag{23}$$

eqn. (21) can be transformed into a much simpler form as

$$\gamma_p\frac{\partial \mathbf{X}_\mathrm{p}(\mathrm{t})}{\partial t} = -k_\mathrm{p}\mathbf{X}_\mathrm{p}(\mathrm{t}) + \tilde{\mathbf{f}}_\mathrm{p}^\mathrm{r}(\mathrm{t}) + \tilde{\mathbf{f}}_\mathrm{p}^\mathrm{act}(\mathrm{t}), \tag{24}$$

where $k_\mathrm{p} = \frac{2kp^2\pi^2}{N}$, where the transformed noise $\tilde{\mathbf{f}}^\mathrm{r}_\mathrm{i}(\mathrm{t})$ follows

$$\begin{aligned}\langle \tilde{\mathrm{f}}^\mathrm{r}_{\alpha,\mathrm{p}}(\mathrm{t}), \tilde{\mathrm{f}}^\mathrm{r}_{\beta,\mathrm{q}}(\mathrm{t'})\rangle &= \frac{\mathrm{k_B T}}{N}\gamma\delta_{pq}\delta_{\alpha\beta}\delta(t-t') \\ \langle \tilde{\mathrm{f}}^\mathrm{r}_{\alpha,\mathrm{p}}(\mathrm{t})\rangle &= 0\end{aligned} \tag{25}$$

and $\tilde{\mathbf{f}}_\mathrm{act}$ is transformed to [11]

$$\begin{aligned}\langle \tilde{\mathrm{f}}^\mathrm{act}_{\alpha,\mathrm{p}}(\mathrm{t})\rangle &= \int_0^N \phi_{pi}\mathrm{f_a}\langle \hat{\mathbf{u}}_i(t)\rangle\, di = 0 \\ \langle \tilde{\mathrm{f}}^\mathrm{act}_{\alpha,\mathrm{p}}(\mathrm{t}), \tilde{\mathrm{f}}^\mathrm{act}_{\beta,\mathrm{q}}(\mathrm{t'})\rangle &= \int_0^N\int_0^N \phi_{pi}\phi_{qj}\mathrm{f_a^2}\langle \hat{\mathbf{u}}_i(t)\cdot\hat{\mathbf{u}}_j(t)\rangle\, didj \\ &= \frac{\mathrm{f_a^2}}{N^2}\int_0^N\int_0^N \cos\left(\frac{\mathrm{ip}\pi}{N}\right)\cos\left(\frac{\mathrm{jq}\pi}{N}\right)e^{-2D_r|t-t'|}\delta_{\alpha\beta} \\ &= \frac{\mathrm{f_a^2}}{2N^2}e^{-2D_r|t-t'|}\delta_{pq}\delta_{\alpha\beta}\end{aligned} \tag{26}$$

The solution to the transformed equation of motion (24) is given as

$$\mathbf{X}_\mathrm{p}(\mathrm{t}) = \mathbf{X}_\mathrm{p}(0)e^{-\frac{p^2}{\tau}t} + \frac{1}{\gamma_p}\int_0^t e^{-\frac{p^2}{\tau}(t-t')}\left(\tilde{\mathbf{f}}^\mathrm{r}_\mathrm{p}(t') + \tilde{\mathbf{f}}^\mathrm{act}_\mathrm{p}(t')\right)dt' \tag{27}$$

where the relaxation time τ_p is

$$\tau_p = \frac{k_p}{\gamma_p} = \frac{k}{\gamma}\left(\frac{p\pi}{N}\right)^2 \tag{28}$$

Now, with cumulant equations (25) and (26), we can derive desired statistical quantities, such as the average bond length L_b and the average radius of gyration Rg, of our active ring polymer using eqn. (27).

Since L_b is the average length between neighboring beads, we have

$$\begin{aligned}\langle L_b^2\rangle &= \left\langle \frac{1}{N}\int_0^N |\mathbf{r}_{n+1}(t)-\mathbf{r}_n(t)|^2\, dn\right\rangle \\ &= \frac{2}{N}\left\langle \int_0^N \mathbf{r}_n^2(t)\, dn + \int_0^N \mathbf{r}_{n+1}\mathbf{r}_n(t)\, dn\right\rangle \\ &= \frac{2}{N}\left\langle 4\sum_{p=even}^{\infty}\sum_{p'=even}^{\infty}\mathrm{X}_p\mathrm{X}'_p\int_0^N \cos\frac{p\pi n}{N}\cos\frac{p'\pi n}{N}\, dn \right. \\ &\qquad \left. -4\sum_{p=even}^{\infty}\sum_{p'=even}^{\infty}\mathrm{X}_p\mathrm{X}'_p\int_0^N \cos\frac{p\pi(n+1)}{N}\cos\frac{p'\pi n}{N}\, dn\right\rangle \\ &= 8\left\langle \sum_{p=even}^{\infty}\mathbf{X}_p\mathbf{X}_p - \sum_{p=even}^{\infty}\mathbf{X}_p\mathbf{X}_p\cos\frac{p\pi}{N}\right\rangle \\ &= 8\sum_{p=even}^{\infty}\langle\mathbf{X}_p\mathbf{X}_p\rangle\left(1-\cos\frac{p\pi}{N}\right) \\ &= 8\sum_{p=even}^{\infty}\langle\mathbf{X}_p\mathbf{X}_p\rangle\sin^2\frac{p\pi}{2N}\end{aligned} \tag{29}$$

where we used eqn. (20) to write out $\mathbf{r}_\mathrm{i}(\mathrm{t})$. Once we know $\langle\mathbf{X}_p(t)\cdot\mathbf{X}_p(t)\rangle$, we can get the averaged bond length in its analytical form.

With some lengthy calculations, we get $\langle \mathbf{X}_p(t) \cdot \mathbf{X}_p(t) \rangle$ as

$$\langle \mathbf{X}_p(t) \cdot \mathbf{X}_p(t) \rangle = \frac{k_b T N}{2k\pi^2 p^2} + \frac{\mathrm{f_a}^2 N^3}{2\pi^2 k} \left(\frac{1}{p^2} \frac{1}{k\pi^2 p^2 + \mathrm{D_r} N^2 \gamma} \right). \tag{30}$$

Plug the this back into eqn. (29), we get

$$\begin{aligned} \langle L_b^2 \rangle &\sim \sum_{p=1}^{\infty} \frac{k_b T N}{2k\pi^2 p^2} \sin^2\left(\frac{p\pi}{2N}\right) + \sum_{p=1}^{\infty} \frac{\mathrm{f_a}^2 N^3}{4\pi^4 k} \left(\frac{1}{kp^4\pi^2 + \mathrm{D_r} N^2 \gamma p^2} \right) \sin^2\left(\frac{p\pi}{2N}\right) \\ &\sim A + B\mathrm{f_a}^2 \\ &\sim A + B\mathrm{Pe}^2 \end{aligned} \tag{31}$$

where the A and B are irrelevant when considering active force. We here comment that the first term is relevant only in ideal passive systems, while the second term is of interest when we are dealing with the active regime. It follows then by absorbing all constants

$$L_b \sim \sqrt{1 + C\mathrm{Pe}^2} \tag{32}$$

As we can see, the bond and spatial distance of beads in the polymer increases as activity increases for an ideal ring. Although a similar derivation is impossible after the inclusion of the Excluded-Volume (EV) interaction, where a series of intriguing phenomena emerge, we note here as Pe becomes large enough, the EV effect becomes irrelevant as bonds get more stretched and the spatial distance of particles enlarges. This, in turn, explains our finding in the main text that bonds get stretched in the high activity regime and thus a tightened knot.

8 Supplementary Movies

- movie_s1.mp4 shows a trefoil knot at Pe = 0
- movie_s2.mp4 shows a trefoil knot at Pe = 20
- movie_s3.mp4 shows a trefoil knot at Pe = 120
- movie_s4.mp4 shows dynamically a trefoil knot at Pe = 120 with knot shrinks smoothly
- movie_s5.mp4 shows a trefoil knot at Pe = 120 with knot shrinks but hindered by bundled structures

References

[1] J. A. Anderson, J. Glaser, and S. C. Glotzer, "Hoomd-blue: A python package for high-performance molecular dynamics and hard particle monte carlo simulations," *Computational Materials Science*, vol. 173, p. 109363, 2020.

[2] J. Stenhammar, D. Marenduzzo, R. J. Allen, and M. E. Cates, "Phase behaviour of active brownian particles: the role of dimensionality," *Soft Matter*, vol. 10, no. 10, pp. 1489–1499, 2014.

[3] S. K. Anand and S. P. Singh, "Conformation and dynamics of a self-avoiding active flexible polymer," *Physical Review E*, vol. 101, no. 3, p. 030501, 2020.

[4] E. Locatelli, V. Bianco, and P. Malgaretti, "Activity-induced collapse and arrest of active polymer rings," *Physical Review Letters*, vol. 126, no. 9, p. 097801, 2021.

[5] K. Kremer and G. S. Grest, "Dynamics of entangled linear polymer melts: A molecular-dynamics simulation," *The Journal of Chemical Physics*, vol. 92, no. 8, pp. 5057–5086, 1990.

[6] B. Marcone, E. Orlandini, A. L. Stella, and F. Zonta, "Size of knots in ring polymers," *Physical Review E—Statistical, Nonlinear, and Soft Matter Physics*, vol. 75, no. 4, p. 041105, 2007.

[7] E. Orlandini, A. L. Stella, and C. Vanderzande, "The size of knots in polymers," *Physical biology*, vol. 6, no. 2, p. 025012, 2009.

[8] J. D. Weeks, D. Chandler, and H. C. Andersen, "Role of repulsive forces in determining the equilibrium structure of simple liquids," *The Journal of chemical physics*, vol. 54, no. 12, pp. 5237–5247, 1971.

[9] M. Caraglio, C. Micheletti, and E. Orlandini, "Stretching response of knotted and unknotted polymer chains," *Physical review letters*, vol. 115, no. 18, p. 188301, 2015.

[10] M. Doi and S. F. Edwards, *The theory of polymer dynamics*, vol. 73. oxford university press, 1988.

[11] R. G. Winkler, A. Wysocki, and G. Gompper, "Virial pressure in systems of spherical active brownian particles," *Soft matter*, vol. 11, no. 33, pp. 6680–6691, 2015.
